# Myopericarditis Mimicking an Acute MI

**DOI:** 10.7759/cureus.51069

**Published:** 2023-12-25

**Authors:** Khurram Arshad, Antoine Egbe Bessong Tabot, Rabia Latif, Ahmad El Gammal, Adam Foglesong

**Affiliations:** 1 Internal Medicine, Corewell Health East, Dearborn, USA; 2 Internal Medicine, Mclaren Flint Hospital, Flint, USA; 3 Cardiology, Corewell Health East, Dearborn, USA

**Keywords:** acute myocardial infarction, elevated cardiac markers, focal st elevation, chest pain, myopericarditis

## Abstract

Chest pain with focal ST elevation in the presence of elevated cardiac markers is usually believed to be secondary to an acute myocardial infarction. Moreover, widespread ST elevation is believed to be a hallmark sign of acute pericarditis. However, we present the case of a young male who presented with chest pain, elevated troponins, and focal ST elevations; however, left heart catheterization showed patent coronary arteries. The patient was treated for acute myo-pericarditis with ibuprofen and colchicine.

This case illustrates the fact that focal ST elevation in a patient with chest pain and elevated markers of cardiac injury is not always secondary to an acute myocardial infarction.

## Introduction

Chest pain represents about 5% of presenting complaints in emergency departments in the USA [1]. Chest pain with ST elevations in contigious leads is usually considered to be acute myocardial infarction (MI) until proven otherwise. However, ST elevations are not pathognomonic to MI; there are other conditions like pericarditis [2], pulmonary embolism, pneumothorax, subarachnoid hemorrhage, hypertrophic and takotsubo cardiomyopathy, aortic dissection, and acute pancreatitis that can also cause ST elevations [3-6]. ST elevation on an electrocardiogram can be the presenting sign of a diagnosis that carries a poor prognosis if not managed promptly. It is, therefore, imperative that we eliminate these potential life-ending differential diagnoses of ST elevations on an EKG while keeping an open mind to other possible diagnoses.

We present a case report of a 39-year-old patient who presented with chest pain and elevated troponins and was found to have ST elevation in V4-V6 on an electrocardiogram, but left heart catheterization did reveal patent coronary arteries. This case report was previously presented as a poster presentation at the American College of Physicians-Michigan Chapter conference in Bellaire, Michigan, on October 21st, 2023.

## Case presentation

A 39-year-old male with no significant past medical history presented to the emergency department (ED) for chest pain that started about three days before he arrived in the ED. Associated symptoms included shortness of breath, but they did not report diaphoresis or any exacerbating or alleviating factors. The chest pain, which radiated down his left arm, was not relieved by aspirin or nitroglycerin, and he did not report any improvement in his symptoms with a change in position. He reported a cough, a runny nose, and myalgias, which had been ongoing for about five days.

Vital signs upon arrival in the ED, blood pressure 138/91; pulse 68; temperature 98.1°F (36.7 °C) (oral); respiratory rate 15; height 180.3 cm (5' 11"); weight 81.6 kg (180 lb); pulse oximetry 97%; body mass index 25.10 kg/m2. On physical examination, the patient was alert, with a Glasgow coma score of 15/15. On the cardiovascular exam, S1 and S2 were present with no rubs, murmurs, or gallops. The initial investigation that was done in the emergency department is mentioned in Table 1. He was also found to be COVID-positive.

**Table 1:** Initial investigations in the emergency department WBC: White blood cells

Investigations	Results	Investigations	Results	Investigations	Results
Troponins*	35.49 ng/ml *	Potassium	4.0 mmol/l	Anion gap	9
COVID-19	Positive	Creatinine	0.71 mg/dl	Bicarbonate	24 mmol/l
WBC	11.9 g/dl	Magnesium	2.1 mg/dl	Chloride	105 mmol/l
Sodium	138 mmol/l	Phosphorus	3.5mg/dl	Calcium	9.2 mg/dl

**Figure 1:**
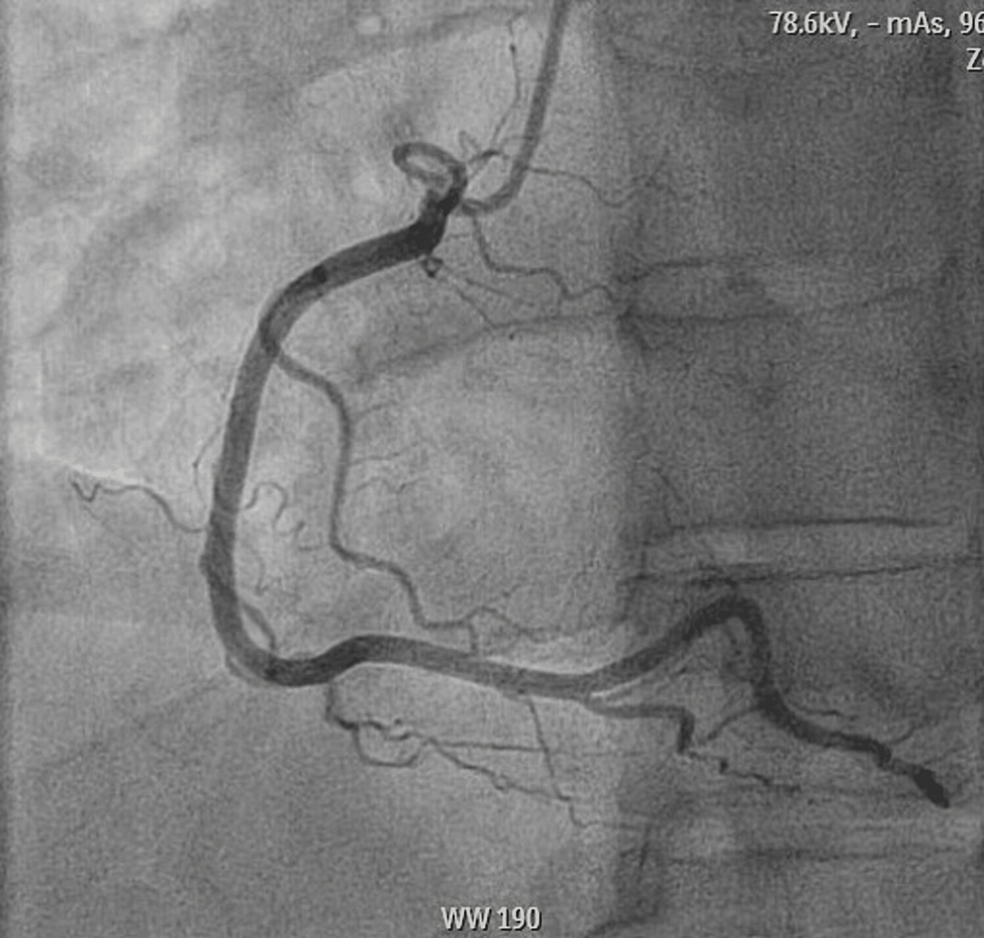
A coronary angiogram shows a patent right coronary artery.

**Figure 2:**
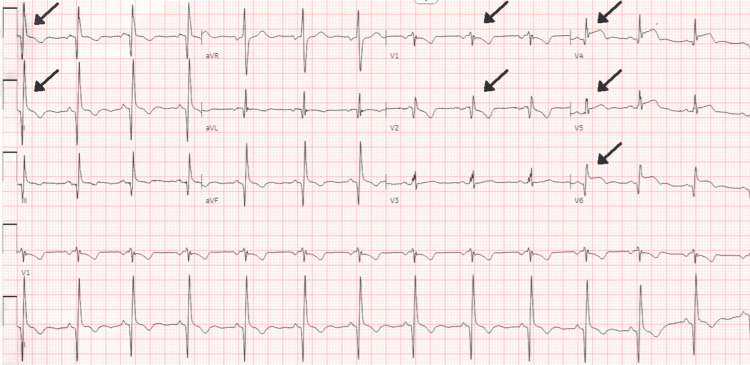
Electrocardiogram (EKG) on arrival to the ED, showing ST elevations in V4, V5 and V6 and T-wave inversions in V1 and V2, suggests an anterior and inferior myocardial infarction.

**Figure 3:**
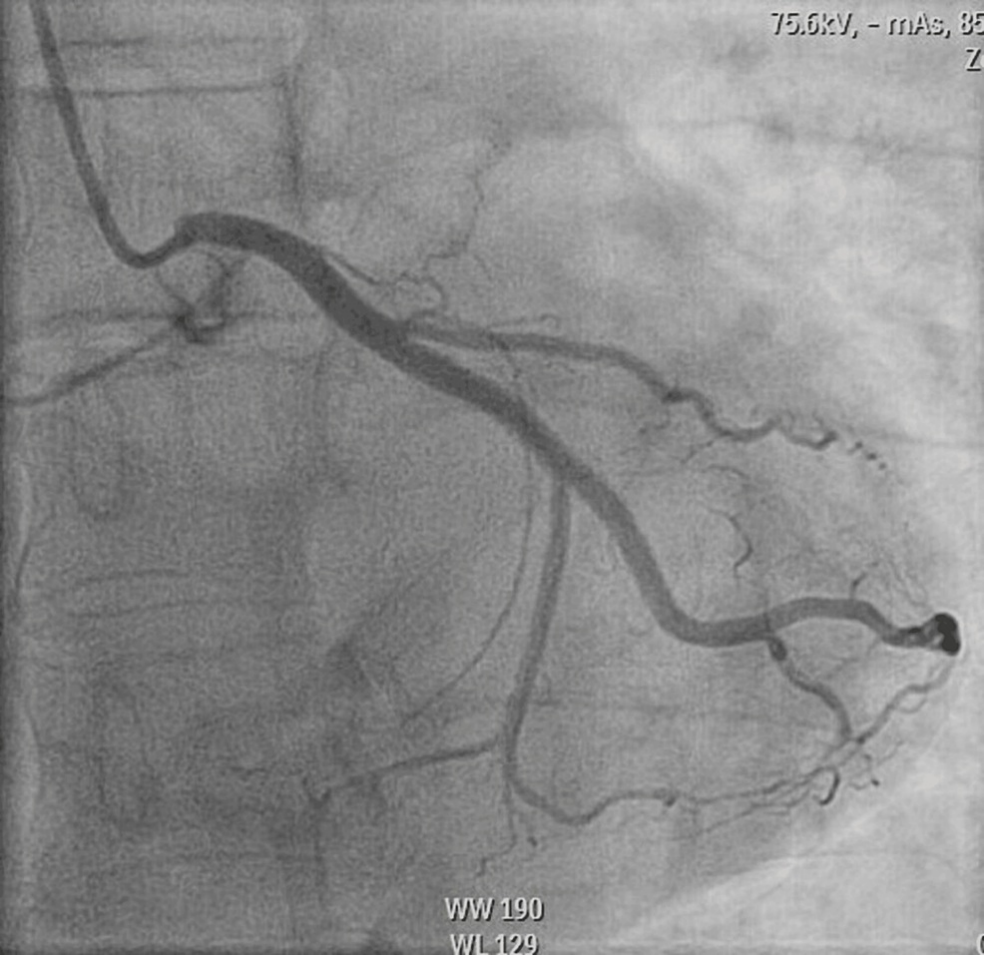
A coronary angiogram shows a patent left circumflex artery.

**Figure 4:**
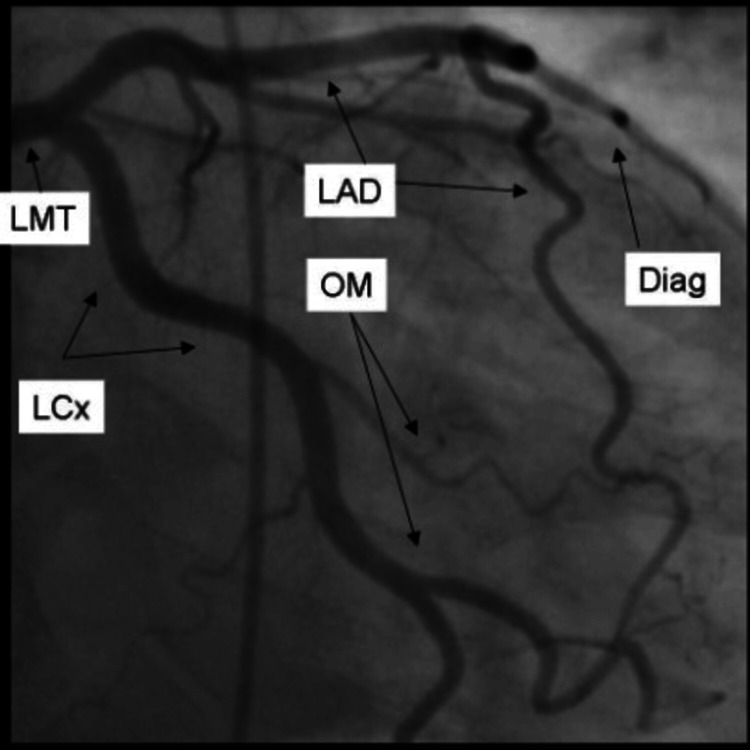
Coronary angiogram showing patent LAD, LCx, and Left main trunk LAD:  Left anterior descending artery, LCx: Left circumflex artery, LMT: Left main trunk

The electrocardiogram (Figure 2) revealed ST elevations in leads V4, V5, and V6. In light of the ECG findings mentioned above, the patient was started on dual antiplatelet therapy and heparin infusion, and he was urgently transported to the catheterization lab for left heart catheterization with possible angioplasty or stenting depending on the results of the coronary angiogram. Left heart catheterization (Figures 1, 3, 4) revealed a very large and patent right coronary artery (RCA), a large caliber and patent left anterior descending artery (LAD), a large caliber and patent left circumflex artery (LCX), a very short and immediate left main artery (LM), a left ventricular ejection fraction of 70%, and a left ventricular end-diastolic pressure of 5 mmHg.

A transthoracic echocardiogram (Video 1) was performed after the left heart catheterization, and it revealed the patient had a left ventricular ejection fraction of 55% with no hypokinetic walls and no valvular abnormalities.

**Video 1:** Transthoracic echocardiogram showing normal wall motion, normal left ventricular ejection fraction, and no valvular abnormalities

**Figure 5:**
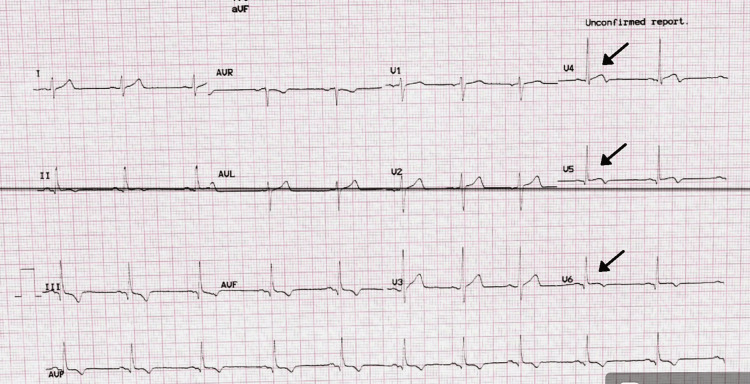
EKG showing resolution of ST-T changes in leads V5, V6 with a partial resolution of the ST elevation lead V4.

A presumptive diagnosis of myo-pericarditis was made, and the patient was started on ibuprofen 600 mg every six hours and colchicine 0.6 mg bid. The already-started dual antiplatelet therapy and heparin infusion were discontinued. Furthermore, day one of the patient’s hospital stay was complicated by a 34-beat run of ventricular tachycardia (VT). Because of the sustained VT, the patient was started on metoprolol 25mg twice daily. By day two of admission, the patient’s chest pain had vanished completely, he was stable, and his oxygen saturation was 96% on room air. He was then moved from the cardiac intensive care unit to the normal acute care unit. The patient was discharged on day three with a scheduled appointment with the cardiologist in two weeks. The EKG (Figure 5) at his second appointment, two months after being discharged from the hospital, is shown below.

## Discussion

Our case highlights the difficulty in differentiating between acute myo-pericarditis and acute myocardial infarction (MI). We had a young patient with chest pain and no risk factors for coronary artery disease, but the focal EKG findings, the elevated troponins, and the excruciating chest pain made the diagnosis of an acute MI very likely.

Myo-pericarditis is an inflammation of the myocardium and the pericardium. The pericardium is a double-walled sac that surrounds the heart and its vessels. The pericardium is made up of the serous visceral layer and the fibrous parietal layer; in between lies the pericardial cavity, which contains pericardial fluid. Pericarditis refers to inflammation of the pericardium; however, the serous visceral layer is juxtaposed with the myocardium. Hence, pericarditis is frequently accompanied by some myocardial involvement. The term myocarditis was coined to refer to a primarily pericarditic syndrome, while the term peri-myocarditis refers to a predominantly myocarditis syndrome [7]. Myopericarditis as a clinical diagnosis is based on the presence of two of the following criteria: pericardial chest pain, which is typically pleuritic in nature and improves when the patient sits up and leans forward; pericardial friction rub, which is a squeaking or scratchy sound best heard with the diaphragm of a stethoscope; new EKG changes, typically widespread ST elevation or depression in the acute phase of pericarditis; and a new or worsening pericardial effusion. Looking at our patient, he met one of the criteria, but the EKG changes were not typical for acute myo-pericarditis. Widespread ST elevation has been reported as the hallmark sign of acute pericarditis, but in the case of our patient, it wasn’t present, hence making the diagnosis more difficult to predict. Electrocardiogram (EKG) changes simply mean inflammation of the epicardium because the parietal pericardium is electrically inert [8].

In developed countries, viral infections are the most common cause of acute myopericarditis, while tuberculosis is the most common cause of myopericarditis in developing countries [9]. The etiology of acute myo-pericarditis can be divided into two main groups: infectious versus noninfectious causes. Infectious causes include i) viruses, for example, adenovirus, coxsackie virus, parvovirus B-19, Epstein-Barr virus, human herpesvirus 6, and cytomegalovirus. ii) bacteria, for example, *Mycobacterium tuberculosis* [10,11], *Coxiella burnetii*, *Borrelia burgdorferi*, rarely *Streptococcus* species, and *Chlamydia* species. iii) Fungal causes include *histoplasma* species [12], *aspergillus* species, *blastomyces* species, and *candida* species. iv) Parasitic causes like *Toxoplasma* species [13] and *Echinococcus* species [14].

The noninfectious causes include the following: i) autoimmune diseases like systemic lupus erythematosus [15,16], Sjogren syndrome, rheumatoid arthritis, Behcet syndrome, Takayatsu arteritis, sarcoidosis, eosinophilic granulomatosis with polyangiitis, and Horton disease, ii) metabolic causes such as uremia [17] and myxedema, iii) traumatic causes such as esophageal perforation and penetrating thoracic injury, iv) Iatrogenic causes such as post-myocardial infarction syndromes, post-pericardiotomy syndromes, post-coronary percutaneous intervention syndromes, and pericarditis post-pacemaker lead insertion. Other causes include drug-related causes, amyloidosis, chronic heart failure, aortic dissection, pulmonary arterial hypertension, and congenital absence of the pericardium [18]. In the case of our patient, he tested positive for COVID-19, and he reported some respiratory symptoms that had begun about 5-7 days before he arrived at the ED. We believe the COVID-19 infection was the most likely cause of myopericarditis in this particular scenario.

We managed our patient with a joint therapy of ibuprofen, a non-steroidal inflammatory drug, and colchicine, known to interfere with several inflammation pathways, which include neutrophil recruitment and adhesion, the RhoA/Rho effector kinase (ROCK) pathway, the tumor necrosis factor-alpha (TNF-α)-induced nuclear factor κB (NF-κΒ) pathway, superoxide production, and inflammasome activation [19]. The management of acute pericarditis depends on the etiology. Idiopathic acute myo-pericarditis, which is the most common per recommendation, is to be managed with aspirin or NSAIDs unless the patient has contra-indications to NSAID utilization. Colchicine is also recommended as an adjunct to prevent recurrences [20-22]. Per the meta-analysis by Samer et al. [20], the utilization of colchicine with NSAIDs for the treatment of acute pericarditis rather than NSAIDs alone will prevent one in four patients who had acute pericarditis from having a pericarditis recurrence. However, in the colchicine for acute pericarditis trial (COPE trial), the number needed to treat was five [22]. In patients who have a contraindication to NSAIDs or aspirin use, low-dose corticosteroids are recommended as the next line of treatment for acute myo-pericarditis. One of the most feared complications of acute myo-pericarditis is a recurrence of the pericarditis. Recurrent myo-pericarditis is managed with NSAIDs or aspirin as first-line therapy, low-dose corticosteroids as next-line therapy, and intravenous immunoglobulin, anakinra, and azathioprine as reasonable alternatives in cases refractory to conventional medical therapy [22-24]. As a last resort, pericardiectomy is an option for recurrent myo-pericarditis, which has been resistant to pharmacologic therapy.

## Conclusions

Acute myo-pericarditis can present with chest pain, focal ST-segment elevations, and elevated cardiac markers. It is always necessary to eliminate an acute myocardial infarction in this scenario. The management of acute myo-pericarditis depends on the etiology. In more economically developed countries, viral infections are the most common cause, while tuberculosis is the most common in less economically developed countries. Acute idiopathic myo-pericarditis is managed with the aid of colchicine and NSAIDs/aspirin.
